# Multiple myeloma can be accurately diagnosed in acute kidney injury patients using a rapid serum free light chain test

**DOI:** 10.1186/s12882-017-0661-z

**Published:** 2017-07-20

**Authors:** Jennifer L. J. Heaney, John P. Campbell, Punit Yadav, Ann E. Griffin, Meena Shemar, Jennifer H. Pinney, Mark T. Drayson

**Affiliations:** 1 Institute of Immunology and Immunotherapy, College of Medical and Dental Sciences, University of Birmingham, B15 2TT, Birmingham, UK; 2 Department for Health, University of Bath, Bath, UK; 3 Department of Renal Medicine, University Hospital Birmingham NHS Foundation Trust, Birmingham, UK; 4 Abingdon Health Ltd., York, UK

**Keywords:** Myeloma, Acute kidney injury, Renal impairment, Serum free light chain, Patient screening

## Abstract

**Background:**

Acute kidney injury (AKI) is common in patients with multiple myeloma (MM). Whether serum free light chain (sFLC) measurements can distinguish between myeloma and other causes of AKI requires confirmation to guide early treatment. A rapid and portable sFLC test (Seralite®) is newly available and could reduce delays in obtaining sFLC results and accelerate diagnosis in patients with unexplained AKI. This study evaluated the accuracy of Seralite® to identify MM as the cause of AKI.

**Method:**

sFLCs were retrospectively analysed in patients with AKI stage 3 as per KDIGO criteria (i.e. serum creatinine ≥354 μmol/L or those on dialysis treatment) (*n* = 99); 45/99 patients had a confirmed MM diagnosis.

**Results:**

The Seralite® κ:λ FLC ratio accurately diagnosed all MM patients in the presence of AKI: a range of 0.14–2.02 returned 100% sensitivity and specificity for identifying all non-myeloma related AKI patients. The sFLC difference (dFLC) also demonstrated high sensitivity (91%) and specificity (100%): an optimal cut-off of 399 mg/L distinguished between myeloma and non-myeloma AKI patients. We propose a pathway of patient screening and stratification in unexplained AKI for use of Seralite® in clinical practice, with a κ:λ ratio range of 0.14–2.02 and dFLC 400 mg/L as decision points.

**Conclusions:**

Seralite® accurately differentiates between AKI due to MM and AKI due to other causes in patients considered at risk of myeloma. This rapid test can sensitively screen for MM in patients with AKI and help inform early treatment intervention.

## Background

Acute kidney injury (AKI) is a common complication of multiple myeloma and has been recommended to be treated as a medical emergency [1]. Renal impairment contributed to nearly a third of deaths that occurred within 60 days of diagnosis in myeloma patients enrolled in UK clinical trials between 1980 and 2002 [2]. Although the survival of myeloma patients presenting with severe renal impairment has significantly improved with modern anti-myeloma therapy the risk of early death remains high [3]. Cast nephropathy (also known as myeloma kidney) is the most common cause of severe renal impairment in patients with myeloma, which occurs due to the aggregation of filtered FLC and Tamm-Horsfall protein in the distal tubule [1]. Nephrotoxic FLCs can cause renal impairment as an early feature of myeloma, prior to other disease-associated organ or tissue damage [4]. AKI secondary to cast nephropathy is only likely when the serum FLC (sFLC) level is ≥500 mg/L [5]. Myeloma-related complications such as hypercalcaemia and dehydration can also cause or exacerbate renal injury [4].

In patients presenting with an AKI where the underlying pathology is unknown, myeloma should be investigated as a potential cause; this requires a robust screening method to be in place [6, 7]. The presence of elevated FLCs and a perturbed sFLC κ:λ ratio is a key marker for detecting plasma cell malignancies and has been shown to be an important indicator of myeloma in the presence of AKI. The first commercially available sFLC assay (Freelite®, The Binding Site, UK) was utilised to determine a κ:λ ratio reference range in patients with renal impairment, without monoclonal gammopathy (renal reference range 0.37–3.1) [8]. This κ:λ ratio range on Freelite® has been demonstrated to provide 100% sensitivity and 99% specificity in identifying multiple myeloma in 41 patients who presented with AKI but has yet to be confirmed in a separate set of myeloma patients [9].

Renal impairment in myeloma has been shown to be reversible in approximately 50% of patients presenting with moderate (plasma creatinine 130–200 μmol/L) to severe (plasma creatinine >200 μmol/L) renal impairment, and recovery of renal function can improve patient survival [10]. Prompt identification and reduction of monoclonal FLCs by anti-myeloma therapy is vital for renal recovery and the prevention of irreversible damage [11–13]. Similarly, in cases where myeloma is not the cause of AKI, it is important to rule this out quickly so that patients can be referred for a kidney biopsy and further clinical investigation. Speed is therefore critical in diagnosing the underlying pathology in AKI. sFLCs assays run on nephelometric or turbidimetric analysers, such as Freelite® and N Latex (Siemens, Germany), are not usually performed in daily batches and often necessitate sending patient samples to specialist laboratories at external sites. This can result in slow turn-around times and delays in receiving patient results. Seralite® (Abingdon Health Ltd., UK) is a lateral-flow test that rapidly quantitates serum κ and λ FLC levels simultaneously in 10 min, which can be easily used as an urgent assay for small numbers of samples in routine hospital laboratories. Seralite® and associated FLC assays using the same anti-FLC reagents have already been shown to perform well in diagnosing and monitoring multiple myeloma in the absence of severe renal impairment [14–17].

The aims of this study were to assess the utility of Seralite® as a screening tool to distinguish between myeloma and non-myeloma related AKI, and provide a reference range for diagnostic purposes in patients presenting with AKI stage 3 where renal impairment was classified as per the Kidney Disease: Improving Global Outcome (KDIGO) criteria [18].

## Methods

### Patients

The present study was a retrospective investigation of patients with AKI stage 3 as per the KDIGO classification (*n* = 99) with stored serum samples available at disease presentation. Patients were included from two separate investigations. Forty five patients were enrolled in the MyEloma Renal Impairment Trial (MERIT: ISRCTN37161699) for patients with newly diagnosed myeloma associated with acute renal failure (creatinine >500 μmol/L, urine output <400 mL/d or requiring dialysis), attributable to myeloma. In the present study, stored serum was analysed from MERIT patients at trial entry. The other patients (*n* = 54) presented with new dialysis-dependent renal failure to the renal unit at the University Hospital Birmingham (UHB). These patients were identified from retrospective analyses of 1090 patients who were admitted to UHB with AKI over a 3 year period. Patients were selected on the basis of stored serum at the time of admission from undergoing initial laboratory screening for a plasma cell disorder. Patients had AKI with an aetiology other than myeloma confirmed through kidney biopsies (*n* = 19) and other clinical investigations (*n* = 35) (non-myeloma related AKI). Healthy individuals (*n* = 91) were also included with serum samples obtained from healthy random donors from the NHS Blood and Transplant service (NHSBT, Birmingham, UK). These donors were anonymous with unknown age and medical history.

### Serum sample analyses

All serum samples underwent analysis for FLCs using Seralite® - a portable lateral-flow test that utilises anti-FLC monoclonal antibodies (Abingdon Health Ltd., Oxford, UK) enabling simultaneous quantification of κ and λ FLC levels (described in full elsewhere) [14]. Healthy donor patient samples were also analysed for creatinine (Roche Hitachi Cobas C501).

### Data analyses

The following sFLC parameters were assessed: absolute κ and λ FLC levels, the κ:λ ratio (κ FLC ÷ λ FLC), FLC sum (κ FLC + λ FLC) and the FLC difference (dFLC; calculated as the involved minus the uninvolved LC in myeloma patients and the higher FLC minus the lower FLC in patients without myeloma and healthy donors). Kruskal-Wallis tests were used to examine differences in FLC parameters measured by Seralite® between groups: AKI patients with myeloma and κ FLC diagnosis, AKI patients with myeloma and λ FLC diagnosis, AKI patients without myeloma and healthy donors. Significant differences were analysed with Dunn’s test post-hoc comparisons. Receiver operating characteristic (ROC) curves were used to assess if sFLC parameters, κ:λ ratio, FLC sum and dFLC, could be used to differentiate between patients with AKI and myeloma and patients with AKI without myeloma. Accuracy was classified using area under the curve (AUC). Best cut-offs were identified using the points on curve closest to the (0, 1); distance was calculated for each observed cut-off point from the curve (distance = √[(1 – sensitivity)^2^ + (1 – specificity)^2^] and the point where the distance was minimum was selected. All analyses were conducted using IBM SPSS statistics version 21.

## Results

### Participants

Myeloma AKI stage 3 patients were aged between 39 and 98 years with a median age of 69 years. Non-myeloma AKI stage 3 patients were aged between 21 and 89 years with a median age of 68 years. There was no significant difference in the age of patient groups nor was there a difference in the proportion of patients aged ≥65 years old: 67% of myeloma patients and 56% of non-myeloma patients were aged ≥65 years. The distribution of sex between the groups did not significantly differ; 56% of myeloma AKI patients and 65% of non-myeloma AKI patients were male. Healthy donors were also included; all with creatinine levels ≤114 μmol/L (mean 81 ± 15 μmol/L).

### Seralite® to differentiate between AKI with myeloma, AKI without myeloma and healthy donors

**Fig. 1:**
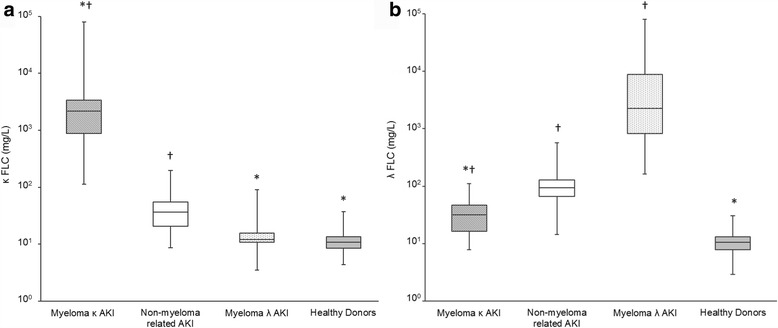
Serum κ FLC (**a**) and λ FLC (**b**) levels analysed using Seralite®. Data is shown for patients with multiple myeloma (with either a monoclonal κ or λ FLC) and acute kidney injury (AKI), patients with AKI without myeloma (non-myeloma related AKI) and healthy donors. Boxes show the interquartile range, with the line indicating the median, and the whiskers show the minimum and maximum. * indicates a significant difference to the non-myeloma related AKI group; † indicates a significant difference to healthy donors; *p* < .01 for all comparisons

Table 1 details full descriptive statistics for all FLC parameters by group. Figure 1 illustrates κ FLC and λ FLC levels in the different cohorts. Significant differences between groups were observed for κ FLC (X^2^ = 132.2, *p* < .001). Myeloma patients (both κ and λ isotype groups) differed to non-myeloma related AKI patients; myeloma κ FLC patients had significantly higher κ FLC levels, and myeloma λ FLC patients had significantly lower κ FLCs, compared with AKI patients without myeloma. Both myeloma κ and non-myeloma related AKI patients had κ FLC levels above healthy donor levels. Significant differences between groups were also observed for λ FLCs (X^2^ = 146.4, *p* < .001). Similarly, both myeloma groups differed to non-myeloma related AKI patients, where myeloma λ patients had higher and κ patients had lower λ FLCs levels than non-myeloma AKI patients. All patient groups had higher λ FLC levels compared with healthy donors. Despite these differences between myeloma AKI patients and AKI patients without myeloma, there was an overlap between the distribution of κ and λ FLC levels between groups.

**Table 1:** Free light chain (FLC) parameters from serum samples analysed using Seralite®

Median (range)	Myeloma AKI κ patients (*n* = 26)	Myeloma AKI λ patients (*n* = 19)	Non-myeloma AKI (*n* = 54)	Healthy donors (*n* = 91)
κ FLC (mg/L)	2193 (113–80,000)	12 (4–91)	36 (9–198)	11 (4–37)
λ FLC (mg/L)	32 (8–110)	2262 (163–80,000)	94 (14–574)	11 (3–31)
κ:λ ratio	81 (3–1103)	0.006 (0–0.090)	0.4 (0.2–1.3)	1 (0.5–3.3)
FLC sum (mg/L)	2215 (131–80,073)	2353 (174–80,017)	134 (26–772)	22 (10–68)
dFLC (mg/L)	2171 (96–79,928)	2189 (152–79,984)	57 (0–376)	3 (0–12)

Patients included multiple myeloma patients with a monoclonal κ or λ FLC and acute kidney injury (AKI), patients with non-myeloma related AKI and healthy donors

**Fig. 2:**
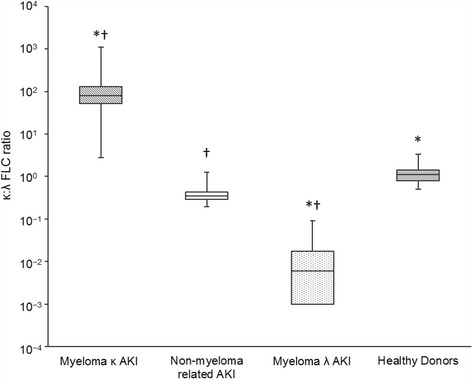
Serum κ:λ FLC ratio analysed using Seralite®. Data is shown for patients with multiple myeloma (with either a monoclonal κ or λ FLC) and acute kidney injury (AKI), patients with AKI without myeloma (non-myeloma related AKI) and healthy donors. Boxes show the interquartile range, with the line indicating the median, and the whiskers show the minimum and maximum. * indicates a significant difference to the non-myeloma related AKI group; † indicates a significant difference to healthy donors; *p* < .05 for all comparisons

The κ:λ ratio revealed complete separation between patient groups (Fig. 2). All myeloma patients exhibited a perturbed ratio outside the healthy diagnostic range (0.5–2.5). There was no overlap in κ:λ ratios between myeloma κ AKI patients (ratio ≥ 2.8) and myeloma λ patients (ratio ≤ 0.009) and non-myeloma AKI patients (ratio 0.2–1.3). The difference between groups was statistically significant (X^2^ = 156.1, *p* < .001); the non-myeloma AKI group had a lower/higher ratio compared with myeloma κ and λ patients with AKI, respectively. Myeloma patients had significantly different ratios compared with healthy donors. Non-myeloma AKI patients had a lower ratio than healthy donors as a result of higher λ FLC levels relative to κ FLC levels. In these patients λ FLC levels were higher than κ levels in 52/54 (96%) patients (in healthy donors λ FLCs were higher than κ in 34/91 (37%) of cases); 48/54 (89%) were below the ratio healthy diagnostic range (0.5–2.5), with the 6 other patients within the range.

**Fig. 3:**
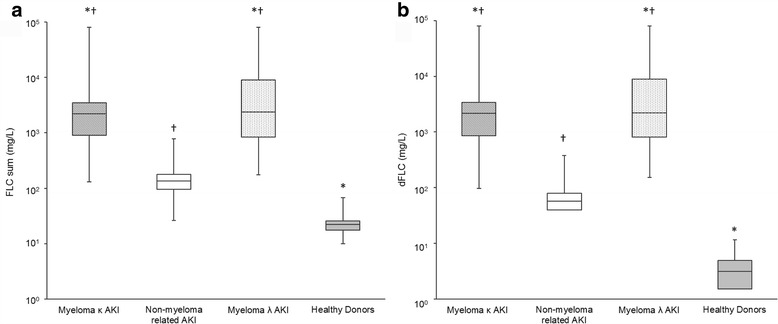
Serum FLC sum (**a**) and FLC difference (dFLC, **b**) analysed using Seralite®. Data is shown for patients with multiple myeloma (with either a monoclonal κ or λ FLC) and acute kidney injury (AKI), patients with AKI without myeloma (non-myeloma related AKI) and healthy donors. Boxes show the interquartile range, with the line indicating the median, and the whiskers show the minimum and maximum. * indicates a significant difference to the non-myeloma related AKI group; † indicates a significant difference to healthy donors; *p* < .01 for all comparisons. FLC sum = κ FLC + λ FLC; dFLC difference = involved minus the uninvolved LC in myeloma patients/maximum FLC minus the minimum FLC in patients without myeloma and healthy donors

The groups had significantly different FLC sums (X^2^ = 157.5, *p* < .001) and dFLC values (X^2^ = 153.1, *p* < .001) (Fig. 3). Both κ and λ myeloma AKI patients had higher FLC sums and dFLC values compared with non-myeloma AKI patients. However, unlike the κ:λ ratio, there was an overlap in the distribution of FLC sums and dFLC values and complete differentiation between groups was not seen with these FLC parameters. All patients had higher FLC sums and dFLC values compared with healthy donors.

### Seralite® sFLC cut-offs to differentiate between AKI patients with myeloma and AKI without myeloma

ROC analyses results for sFLC parameters to identify myeloma in AKI patients are presented in Table 2. The κ:λ ratio gave an AUC of 1.0 indicating this parameter was able to classify myeloma from non-myeloma AKI patients with perfect accuracy. Myeloma κ patients always had higher ratios and myeloma λ patients always lower ratios compared to non-myeloma AKI patients resulting in no false positives/false negatives. Accordingly, this sFLC parameter provided 100% sensitivity and 100% specificity for differentiating between myeloma and non-myeloma related AKI. The optimal cut-offs were 0.14 to identify λ patients and 2.02 to distinguish κ patients. These values can be used to inform an adapted renal reference range for use with this test in patients presenting with AKI stage 3. The FLC sum and dFLC also provided a high level of accuracy for identifying myeloma from non-myeloma AKI. AUCs of .97 and .99 were observed for the FLC sum and dFLC, respectively. The best-cut-offs were 528 mg/L for FLC sum (84% sensitivity, 94% specificity) and 399 mg/L for dFLC (91% sensitivity, 100% specificity).

**Table 2:** Serum FLC parameters to differentiate patients with and without myeloma in patients presenting with AKI

	AUC	Best cut-off	Sensitivity	Specificity
Ratio: myeloma κ	1.0 (95% CI 1–1, *p* < .001)	2.02	100%	100%
Ratio: myeloma λ	1.0 (95% CI 1–1, *p* < .001)	0.14	100%	100%
FLC sum	0.97 (95% CI .94–.1.0, *p* < .001)	528 mg/L	89%	94%
dFLC	0.99 (95% CI .97–1.0, *p* < .001)	399 mg/L	91%	100%

Data is shown ROC analyses for the κ:λ ratio, FLC sum and FLC difference (dFLC) measured using Seralite®. Area under curve (AUC, with 95% confidence intervals [CI]) was calculated and the best cut-off point identified with associated sensitivity and specificity are reported for each FLC parameter

## Discussion

This study assessed whether a new portable sFLC test could serve as a rapid screening tool for myeloma in patients presenting with AKI. Seralite® was able to provide differential diagnosis between myeloma and non-myeloma related AKI. The ROC analyses showed with a 100% certainty that myeloma κ AKI patients would have a higher κ:λ ratio, and myeloma λ AKI patients would have a lower κ:λ ratio, than non-myeloma AKI patients. The κ:λ ratio discriminated between patients with perfect accuracy with no false positives or false negatives with optimal cut-offs of 0.14 and 2.02; this represents an appropriate reference range to apply when using Seralite® in patients with severe renal impairment. The FLC sum and dFLC also provided a high level of accuracy for identifying myeloma in these patients, with optimal cut-offs of 528 mg/L for FLC sum and 399 mg/L for dFLC. The dFLC offered the best combination of sensitivity (91%) and specificity (100%). Although absolute levels of κ and λ FLCs were significantly different between groups there was overlap in the distribution between myeloma and non-myeloma patient results. This is consistent with previous findings where only the κ:λ FLC ratio showed very good sensitivity for myeloma cast nephropathy [19].

Importantly, the portable device offered the same level of diagnostic performance previously reported for conventional laboratory-based testing. The published renal reference for the κ:λ ratio using Freelite® (0.37–3.1) is associated with 100% sensitivity and 99% specificity for detecting nephrotoxic monoclonal FLCs in 41 myeloma patients with AKI [9]. The other commercially available method of sFLC quantitation, N Latex, currently has no published validation of sFLC measurement in patients with myeloma and AKI. However, evaluation has been carried out with N Latex in patients with renal impairment (without myeloma) where, unlike Seralite® and Freelite®, an adapted κ:λ ratio renal reference range was found not to be required [20]. Good clinical concordance has been shown between the different sFLC methods previously [14, 15, 17]. However, it is essential to remember that assays cannot be used interchangeably as they quantitate sFLC levels in different ways, particularly monoclonal FLCs [14, 15, 20, 21]. Users need to ensure consecutive samples from individual patients are compared using the same assay, taking into account the specific healthy and renal reference limits provided by the different tests.

It is estimated that 50% of patients with myeloma-related AKI initially present to nephrology services [22]. sFLC laboratory assays have made important progress in reducing delays associated with other methods of monoclonal FLC testing (serum and urine protein electrophoresis, immunofixation electrophoresis) [9]. However, FLC nephelometric and turbidimetric assays are not available in all hospitals, often necessitating sample dispatch to specialised laboratories. When using automated analysers, samples are routinely run in batches and thus testing may not be carried out every day. This can lead to sample turn-around times of days to weeks and cause delays that may jeopardise the chance of renal recovery. Seralite® accelerates the processing of urgent samples and it could be used to generate results in 10 min by any hospital that operates a 24 h laboratory service. Early diagnosis and treatment intervention in myeloma is central to disease-free survival [2, 23] but is particularly important in the context of myeloma and AKI. Swift treatment intervention to reduce monoclonal FLCs and halt injury is essential: a 60% reduction in FLCs by day 21 has been associated with recovery of renal function for 80% of the population [11]. Seralite® identification of abnormal sFLC levels could enable quicker treatment initiation and in turn potentially improve renal recovery rates in myeloma AKI patients. Further, by quickly identifying a high probability of cast nephropathy patients may be spared the risk of a renal biopsy.

**Fig. 4:**
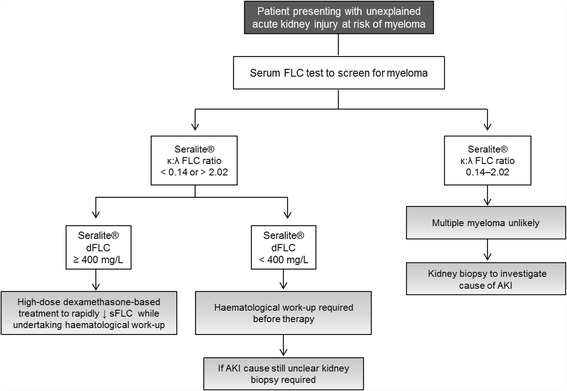
Proposed screening pathway for myeloma in unexplained acute kidney injury (AKI) using Seralite® in patients at risk of myeloma. Screening of individual patients should be informed by risk factors such as age (> 60 years old) or other clinical features where myeloma could be suspected. The κ:λ ratio can be used to screen for myeloma and the FLC difference (dFLC – defined as the maximum FLC level minus the minimum FLC level) can be used to aid subsequent decisions for clinical management. If an abnormal κ:λ ratio is identified using the renal reference range of 0.14–2.02, a dFLC ≥400 mg/L can be used to indicate a high likelihood of cast neuropathy and stratify patients for consideration of immediate therapy to reduce serum FLC levels

Screening pathways have been proposed for myeloma in AKI based upon Freelite® sFLC levels [5, 13]. In these algorithms, when patients have a κ:λ ratio outside the renal reference range, a sFLC level ≥ 500 mg/L on Freelite® indicates a high probability of myeloma cast nephropathy. Consequently, a renal biopsy may not be necessary [24]. High-dose dexamethasone can induce up to a 100-fold reduction of sFLCs within 14 days: immediate administration is crucial in myeloma cast neuropathy and is often started prior to deciding a full programme of induction therapy [12, 13]. Early treatment with dexamethasone could be given to patients who are identified as having a high chance of cast nephropathy via Seralite®. In Fig. 4 we make suggestions for the use of Seralite® in clinical practice for patient screening and stratification in patients with unexplained AKI who are thought to be at risk of myeloma. Using this pathway, the κ:λ ratio could be used to identify monoclonal FLC and the dFLC to aid subsequent decisions for clinical management. If an abnormal κ:λ ratio (< 0.14 or >2.02) is identified, a dFLC threshold of ≥400 mg/L can be used to confirm a high likelihood of cast neuropathy. We recommend patients with an abnormal ratio and a dFLC level of ≥400 mg/L should be considered for immediate treatment with high-dose dexamethasone (guided by haematology and nephrology teams), while concurrently expediting additional laboratory and clinical investigations to confirm myeloma diagnosis, and a renal biopsy is not indicated. Due to the high specificity observed in this study, it is extremely unlikely that using these criteria would result in an AKI patient without myeloma being administered high-dose dexamethasone. As a result of the high sensitivity of this test, it can be expected that missing myeloma patients using sFLC screening would occur only in rare circumstances. In the case of a dFLC <400 mg/L on Seralite®, caution should be taken, awaiting haematological results before considering initiating dexamethasone therapy; if haematological investigations remain inconclusive a biopsy may be required to establish the cause of AKI. For all patients where an abnormal ratio is identified, irrespective of the observed sFLC level, a comprehensive haematological work-up is required urgently. Full anti-myeloma therapy should certainly not be considered until the diagnostic criteria for multiple myeloma, including a bone marrow biopsy, have been fulfilled.

Myeloma usually only accounts for a few percent of new AKI stage 3 cases and so it remains necessary to test the sensitivity and specificity of Seralite® in a population where the proportion of myeloma cases is close to that, rather than nearly half as in this study. A further limitation of the present investigation is the retrospective study design. Although serum samples were collected at the time of myeloma presentation/screening for myeloma, sFLC testing by Seralite® was carried out later. Findings should be confirmed in a prospective study in clinical practice with ‘real time’ Seralite® testing when patients present with AKI. Prospective studies are also required to refine criteria for screening individual patients and assess the health economics of screening. While sFLC screening across all patients with AKI where the cause is unknown may be the ideal to ensure potential myeloma patients are not missed, this approach is not feasible or indeed necessary based upon the expected prevalence of myeloma: here, out of 1090 patients admitted to the renal unit at UHB, 4% had myeloma. Consequently, patient subgroups most appropriate for screening based on risk of myeloma need to be identified. Myeloma most commonly occurs in individuals aged over 60 (80% of myeloma patients in the present study) and this would be a simple criterion to initially stratify patient screening. Age and other clinical factors should be examined to further develop screening strategies for myeloma in AKI.

In a previous investigation of myeloma patients presenting with AKI at diagnosis who underwent a renal biopsy, the principal pathology in nearly 90% of patients was cast nephropathy as a consequence of excess and nephrotoxic monoclonal FLCs [25]. Therefore, myeloma patients with severely reduced kidney function present with high sFLC levels as the direct cause in almost all cases. Alternatively in mild to moderate renal impairment in myeloma, sFLCs may only be a small contributing factor and more likely triggered by myeloma related co-morbidities (dehydration, hypercalcaemia and use of non-steroidal anti-inflammatory drugs) [12]. Unrelated co-morbidities may also play a role: in the age population from which myeloma arises approximately 25% of individuals have mild to moderate renal impairment linked to age-related morbidities, such as hypertension, cardiovascular disease and diabetes [26–28]. The relationship between sFLC levels assessed by Seralite® and different degrees of renal impairment in myeloma requires exploration in separate studies. Further, the sensitively of Seralite® and the suitability of the renal reference range in monoclonal gammopathy of renal significance (MGRS) would need to be independently validated in these disorders that do not meet the criteria for multiple myeloma.

## Conclusions

Serum FLC quantitation via Seralite® could be used as an effective screening tool for myeloma in patients presenting with AKI stage 3. The FLC ratio sensitively and specifically distinguishes between patients with AKI and myeloma or AKI attributable to other causes. Seralite® is easily applicable to testing urgent samples in routine hospital laboratories and therefore could be used to speed up the diagnosis of myeloma in AKI patients, in whom prompt intervention is vital to provide the best chance of renal recovery and improve patient prognosis.

## Abbreviations

AKI: acute kidney injury
AUC: area under curve
FLC: free light chain
KDIGO: (Kidney Disease: Improving Global Outcome)
MM: multiple myeloma
ROC: receiver operating characteristic
sFLC: serum free light chain
